# The oriented and patterned growth of fluorescent metal–organic frameworks onto functionalized surfaces

**DOI:** 10.3762/bjnano.3.66

**Published:** 2012-08-02

**Authors:** Jinliang Zhuang, Jasmin Friedel, Andreas Terfort

**Affiliations:** 1 Institute for Inorganic and Analytical Chemistry, University of Frankfurt, Max-von-Laue-Str. 7, D-60438 Frankfurt, Germany

**Keywords:** electron-beam lithography, irradiation-promoted exchange reaction, microcontact printing, radiation-induced nanostructure, self-assembled monolayer, surface-attached metal–organic framework

## Abstract

A metal–organic framework (MOF) material, [Zn_2_(adc)_2_(dabco)] (adc = anthracene-9,10-dicarboxylate, dabco = 1,4-diazabicyclo[2.2.2]­octane), the fluorescence of which depends on the loading of its nanopores, was synthesized in two forms: as free-flowing nanocrystals with different shapes and as surface-attached MOFs (SURMOFs). For the latter, we used self-assembled monolayers (SAMs) bearing functional groups, such as carboxylate and pyridyl groups, capable of coordinating to the constituents of the MOF. It could be demonstrated that this directed coordination also orients the nanocrystals deposited at the surface. Using two different patterning methods, i.e., microcontact printing and electron-beam lithography, the lateral distribution of the functional groups could be determined in such a way that the highly localized deposition of the SURMOF films became possible.

## Introduction

Metal–organic frameworks (MOFs) are a fascinating class of organic–inorganic hybrid materials with nanometer-sized pores. The size and density of the pores renders these materials with extraordinary large free volumes and inner surfaces, which are accessible by guest molecules. Based on this, MOFs have already demonstrated their potential for gas storage/separation [1], heterogeneous catalysis [2], molecular recognition [3], and sensing [4]. Some of these applications, such as gas storage, require the bulk preparation of the materials, what is typically performed by solvothermal synthesis at high temperatures [5–6]. For more sensitive materials, the interdiffusion method, in which the initially separated reactants slowly diffuse towards each other, is also often used [6–8]. Both procedures have the advantage that relatively large crystals can be obtained, which may be suitable for single crystal X-ray diffraction. For many other applications, such as sensing, these crystals often are too large, since the path lengths for the guest molecules within the nanochannels become too long for a fast response.

Thus, nanoscale MOFs have attracted great attention for sensing purposes, but also for bioimaging and biomedical applications, such as nitric oxide (NO) storage and drug delivery [9]. Several strategies have been developed to obtain control over the size and morphology of the MOF crystals, such as microwave heating [10–11], ultrasonic synthesis [12–13], microemulsions [14–15], or solvent-triggered precipitation [16–17]. Nanoscale MOFs with various morphologies, (e.g., nanospheres [16,18–20], nanocubes [21], nanorods [14,22], nanowheels [23], and hierarchical spheres [22]) have been synthesized [24].

For sensoric applications in particular, the nanoscale MOF should be immobilized at specific locations on surfaces rather than being a free-flowing powder, to facilitate the read-out of their response. Thus, the spatially and morphologically controlled growth of MOFs, in the form of small crystals or films on specific surfaces, gains significant importance [25–27]. Several methods have been developed to control the growth of such surface-attached MOFs (so-called SURMOFs) on various substrates [28–35]. To adjust the surface chemistry of such substrates, self-assembled monolayers (SAMs) are a powerful tool due to the flexibility regarding the functional groups that they expose, which in turn permit a remarkable control over the growth of SURMOFs. In particular, by using a step-wise layer-by-layer procedure, it has been demonstrated that SAMs cannot only control the spatial deposition of MOF films, but also determine the crystallographic orientation within the films [25,29,31].

**Figure 1:**
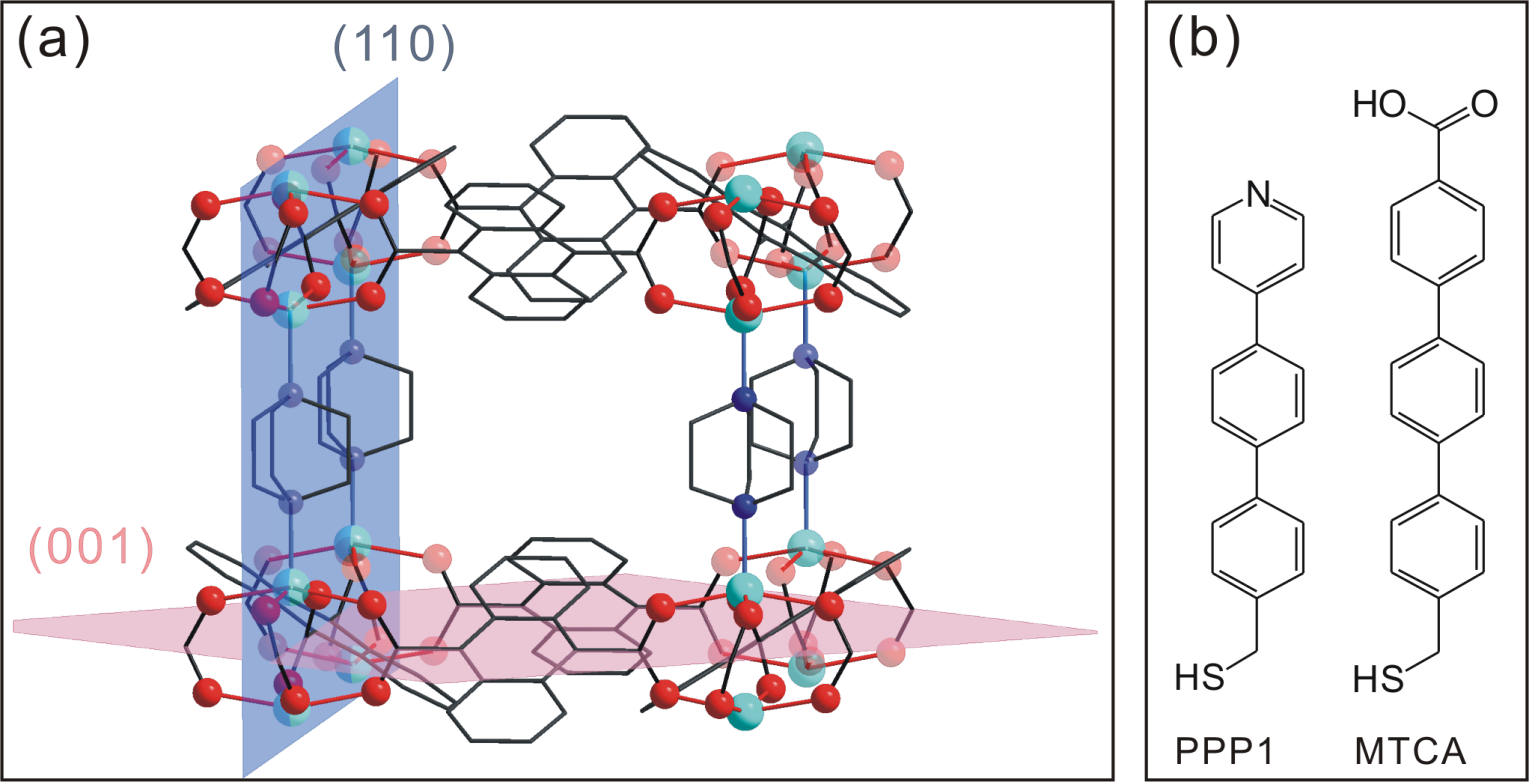
(a) The crystallographic cell of [Zn_2_(adc)_2_(dabco)]. The directionality of the attachment of carboxylate and monodentate Lewis bases (dabco/pyridine) determines the two crystallographic planes given in the scheme. (b) SAM-forming molecules used in this study.

MOFs based on large π-conjugated molecules are expected to be useful optical materials, e.g., as sensors, photocatalysts, or electroluminescent devices [36–37]. In this paper, we describe a rapid route to synthesize photoluminescent MOF nanocrystals at room temperature, and the growth of highly orientated and patterned SURMOFs by using SAMs as a template. We have chosen anthracene-9,10-dicarboxylate (adc) as an organic linker to grow a tetragonal MOF, [Zn_2_(adc)_2_(dabco)] (dabco = 1,4-diazabicyclo[2.2.2]­octane), since anthracene-based compounds show interesting luminescent properties, such as photoluminescence and electroluminescence [36,38–39]. In order to obtain highly orientated SURMOFs, we used two SAMs of very high structural quality: The COOH-terminated SAM was formed from 4′-(mercaptomethyl)-terphenyl-4-carboxylic acid (MTCA, see Figure 1b) [40] and the monodentate Lewis base one was formed from (4′-(pyridin-4-yl)-[1,1′-biphenyl]-4-yl)methanethiol (PPP1, see Figure 1b) [41]. Similar to other M_2_L_2_P (M = Cu, Zn; L = benzene­1,4-dicarboxylate (bdc), tetrafluorobenzene-1,4-dicarboxylate (F_4_bdc), naphthalene­1,4-dicarboxylate (ndc); P = dabco, 4,4′-bipyridine (bipy)) type SURMOFs [42], two different principal growth directions are expected on MTCA and PPP1 surfaces, which correspond to the directionality of the attachment, either of carboxylate ([110] direction) or of pyridyl groups ([001] direction) to the [Zn_2_(adc)_2_(dabco)] crystallographic cell (Figure 1a).

## Results and Discussion

### Synthesis of [Zn_2_(adc)_2_dabco] nanocrystals at room temperature

**Figure 2:**
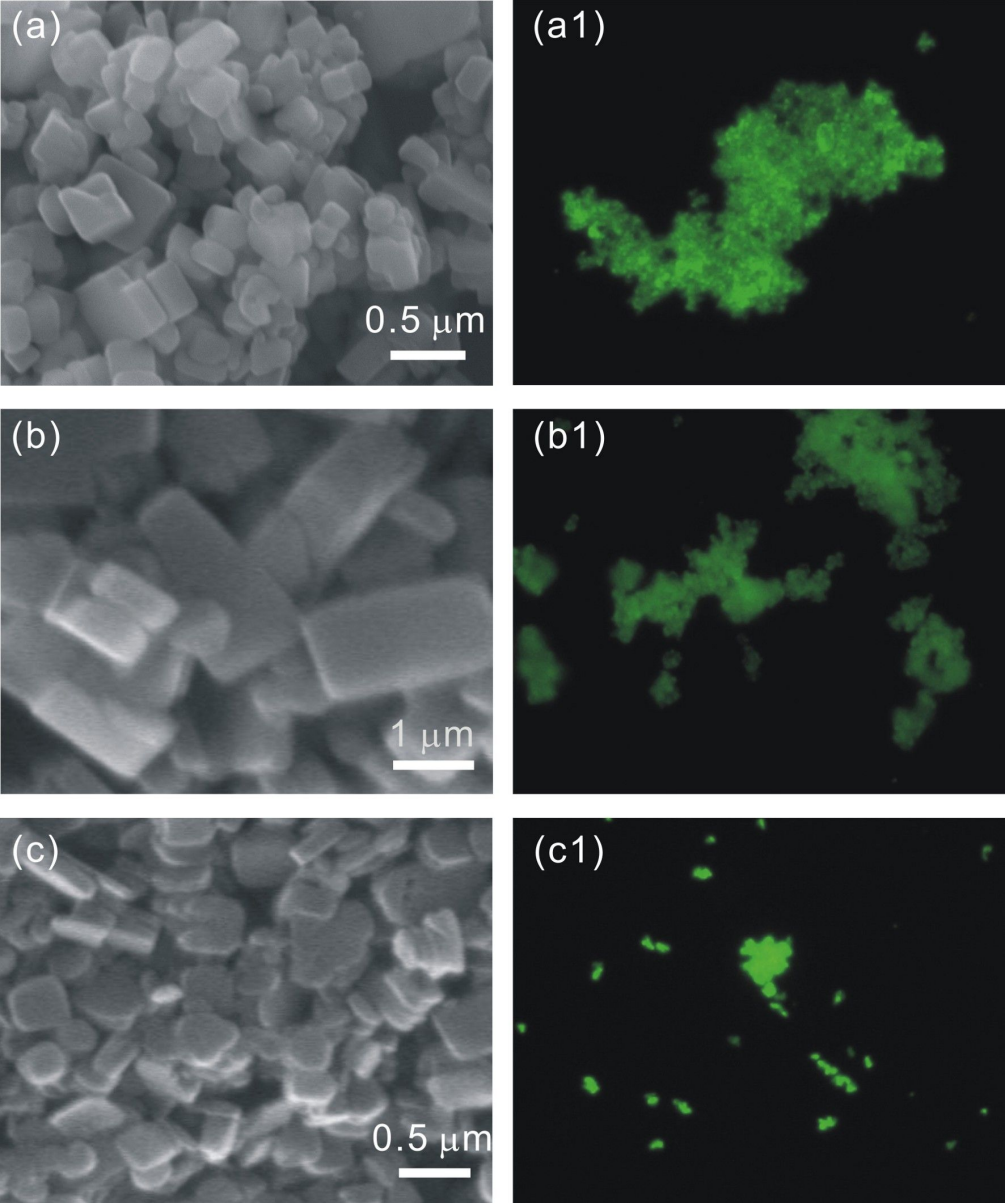
SEM images (a–c) and epifluorescence images (a1–c1) of Zn-MOF nanocrystals synthesized at various concentrations and ratios of the precursors at room temperature. (a) 50 mM of Zn–adc and 50 mM of dabco; (b) 50 mM of Zn–adc and 25 mM of dabco; (c) 25 mM of Zn–adc and 12.5 mM of dabco.

For reference purposes, bulk [Zn_2_(adc)_2_(dabco)] crystals were synthesized in accordance to the literature procedure [36]. This procedure was then varied to explore the possibility of nanocrystal fabrication. Thus various concentrations and ratios of the precursors were used to evaluate the influence on the crystal size and appearance. Figure 2 depicts [Zn_2_(adc)_2_(dabco)] crystals obtained under three different conditions. When an equimolar solution of Zn(NO_3_)_2_ and H_2_adc (”Zn–adc”, 50 mM each in *N*,*N*-dimethylformamide) was mixed with a 50 mM solution of dabco in methanol, cuboid crystals with a size of 200–500 nm became visible in scanning electron microscopy (SEM) images (Figure 2a).

**Figure 3:**
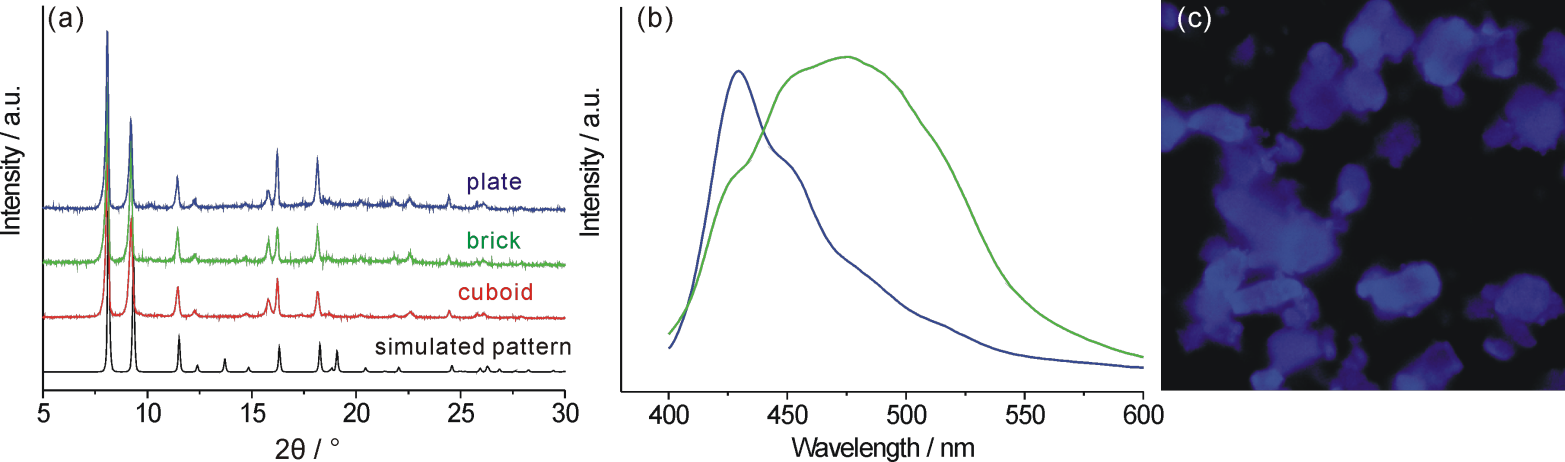
(a) Powder diffractograms of the nanocrystalline products (cuboid (50 mM Zn–adc/50 mM dabco); brick-like (50 mM Zn–adc/25 mM dabco), and plate-like (25 mM Zn–adc/12.5 mM dabco)) in comparison with the one simulated from [Zn_2_(adc)_2_(dabco)] single-crystal data. The minor differences between the powder XRD patterns and the simulated one may result from different guests in the MOF pores. (b) Solid-state emission spectra of as-synthesized [Zn_2_(adc)_2_(dabco)] nanocrystals (loaded with solvent molecules such as DMF, green curve) and in the dried bulk form (blue curve). (c) Fluorescence image of a DMF-free sample. Excitation wavelength was 365 nm.

However, when the concentration of dabco in methanol was reduced to 25 mM, brick-like [Zn_2_(adc)_2_(dabco)] crystals of 1–2 µm were obtained. By maintaining the ratio of Zn/dabco at 2/1 but reducing the concentrations of both precursors to 25 mM (Zn–adc) and 12.5 mM (dabco), respectively, the morphology of the crystals varied again and we obtained plate-like nanocrystals with a size of 300 nm, as shown in Figure 2c. Powder X-ray diffraction (PXRD) studies showed that all the nanocrystals of [Zn_2_(adc)_2_(dabco)] are crystalline and of the same polymorph as described in the literature [36], confirmed by the good agreement of the determined diffractograms with the one simulated from reported crystallographic data (Figure 3a, lowest trace) [36].

Since the distances between the anthracene moieties in the [Zn_2_(adc)_2_(dabco)] framework are large enough to preclude electronic interactions, the absorption and emission spectra should resemble those of molecularly dispersed anthracene, e.g., in solutions. Thus a blue fluorescence would be expected. Instead, as can be seen in the right column of Figure 2, all the nanocrystals show green fluorescence when exposed to UV light. The respective fluorescence spectrum was determined to be quite broad with a maximum at 475 nm (Figure 3b, green curve). According to Tanaka et al. [36] this shift results from the interaction of electron-donor molecules with the anthracene units, and can be used to detect certain analytes, such as *N*-methylaniline or *N*,*N*-dimethylaniline. In our case the *N*,*N*-dimethylformamide (DMF), which was used as a solvent, interacted with the anthracene π-system of the host and caused the observed red shift. This could be easily demonstrated by removing the solvent from the framework by heating under vacuum, which changed the fluorescence of the material to blue (Figure 3c). The respective solid-state emission spectrum of the solvent-free [Zn_2_(adc)_2_(dabco)] crystals (Figure 3b, blue) displays an emission maximum at 429 nm with a vibrational band at 453 nm, similar to the emission from monomeric anthracene [36,43]. This behaviour shall be used as a sensoric principle in future projects.

### Controlled growth of [Zn_2_(adc)_2_dabco] on SAM-functionalized surfaces

**Figure 4:**
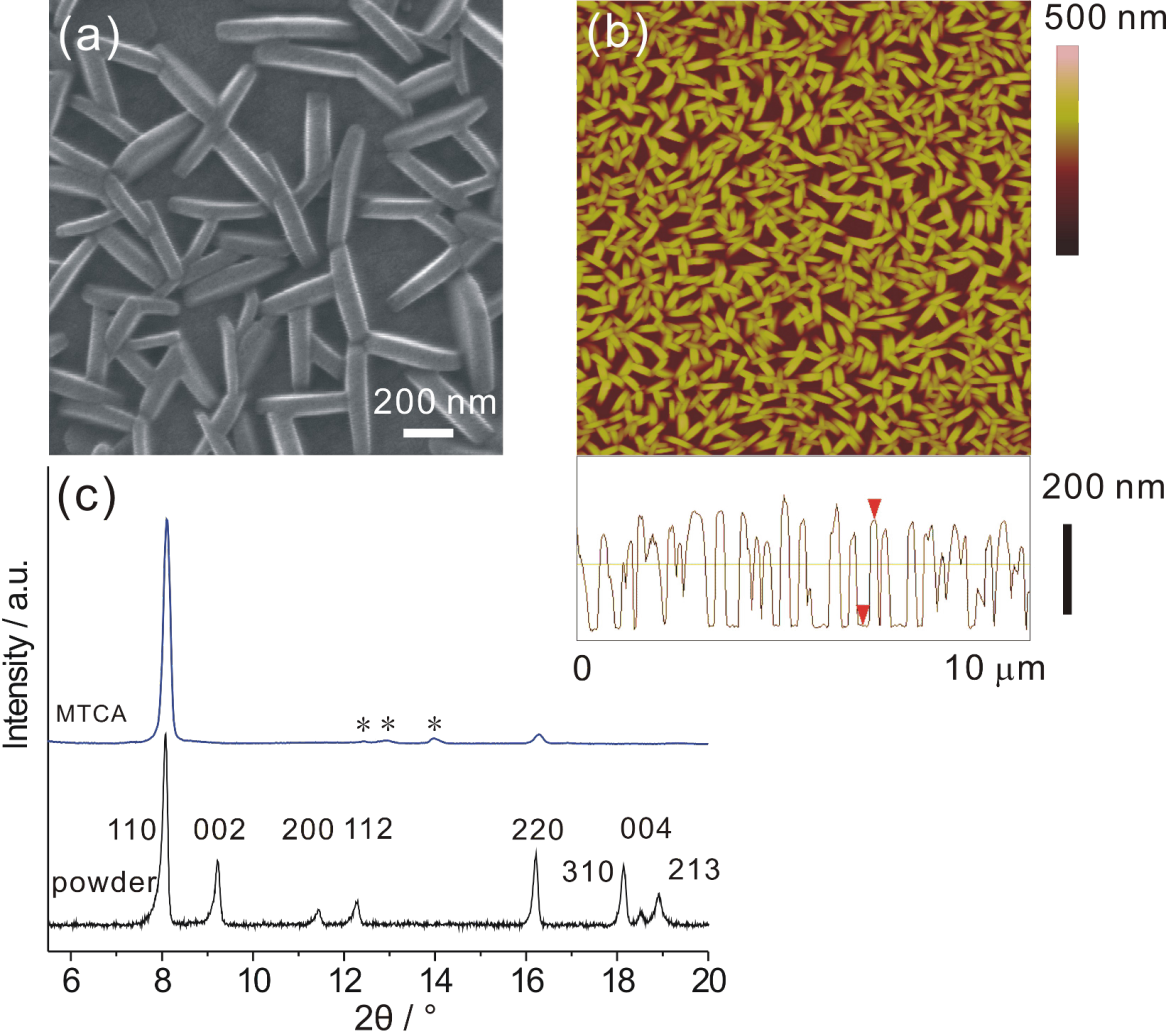
Characterization of [Zn_2_(adc)_2_(dabco)] grown on a MTCA surface at 15 °C after 45 cycles by using the layer-by-layer deposition method. (a) High-magnification SEM image; (b) AFM image and cross-sectional profile; (c) XRD patterns of SURMOF and powder sample. The asterisks denote signals from the background.

As mentioned before, many applications rely on the attachment of the active materials to surfaces [25–26,44]. For the formation of SURMOFs, several strategies exist, such as direct growth/deposition from solvothermal mother solutions [28,30], electrochemical deposition [33], gel-layer deposition [35], spin-coating deposition from a precursor solution [17,45], Langmuir–Blodgett based layer-by-layer method [34,46], and direct step-wise layer-by-layer growth [29,31,44,47–48]. Of these, the latter method is particular suitable, since it is easily performed and provides very good control over the amount of material being deposited. For this deposition technique, essentially the substrate is alternately exposed to a solution containing the metal source and a solution containing the organic linker(s), with purging steps in between. In our case, a solution of zinc acetate in ethanol (1 mM) acted as the metal source, and the ligands were deposited from an equimolar H_2_adc/dabco mixture (0.1 mM each, also in ethanol). When a MTCA-functionalized substrate was used for the deposition at 15 °C, after 45 cycles, uniform plate-like [Zn_2_(adc)_2_(dabco)] nanocrystals with high density on the gold surface could be observed in the SEM and atomic force microscopy (AFM) images (Figure 4).

The thickness and the height of the nanoplates could be estimated from these images and the AFM cross-sectional profile to be around 75 nm and 230 nm, respectively. More interestingly, the nanoplate crystals show a highly vertical alignment at the MTCA surface hinting at a preferred crystallographic orientation. Out-of-plane surface X-ray diffraction (SXRD, Figure 4c) supports this assumption, since only two diffraction peaks at 2θ = 8.1 and 16.2° were observed, which can be assigned to the reflections of the (110) and (220) planes according to the powder XRD pattern of [Zn_2_(adc)_2_(dabco)]. The [110] orientation of the SURMOF is in agreement with the expectation deducible from the crystal structure: The surface carboxylate groups replace, e.g., the leftmost carboxyl groups in Figure 1a, directing the (110) plane (blue) parallel to the substrate surface.

**Figure 5:**
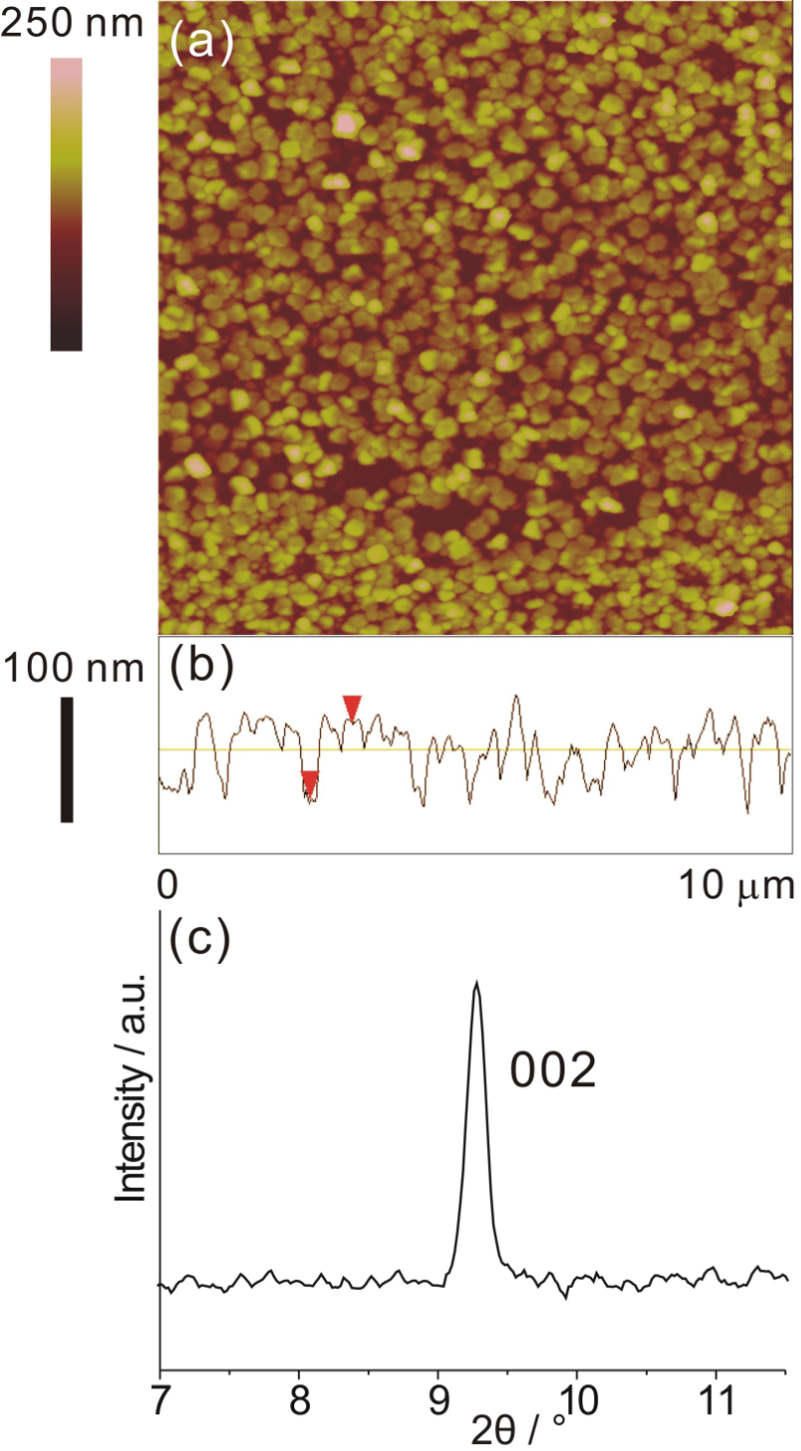
[Zn_2_(adc)_2_(dabco)] grown on a PPP1 surface at 60 °C after 45 cycles. (a) AFM image; (b) cross-sectional profile; (c) SXRD pattern of the range in which the (110) and the (002) reflections are expected.

In contrast, the growth of [Zn_2_(adc)_2_(dabco)] on a SAM with monodentate Lewis base headgroups capable of coordinating to the apical sites of the Zn_2_ units should lead to a [001] orientated SURMOF, in analogy to the observations made for the isoreticular [Cu_2_(ndc)_2_dabco] SURMOF [42]. To test this, we employed the pyridine-terminated PPP1 SAM as a substrate. Again plate-like nanocrystals with a thickness of about 60 nm formed after 45 cycles, as shown in Figure 5a. However, this time the nanoplates were lying on the PPP1 surface. That this morphological change is in fact correlated to a different crystallographic orientation can be clearly seen from the SXRD pattern: Only the diffraction peak corresponding to the (002) planes at 2θ = 9.2° became visible, revealing that in this case the SURMOF was oriented along the [001] direction.

It is noteworthy that the deposition temperature is a crucial factor to achieve a completely orientated SURMOF growth. Thus, for the MTCA surface, the perfect [110] orientation was obtained at 15 °C, while on the PPP1 SAM a complete [001] orientation could only be attained at 60 °C. This temperature effect is now under investigation and is not part of the current work.

### Fabrication of patterned [Zn_2_(adc)_2_dabco] films by microcontact printing and electron-beam lithography techniques

**Figure 6:**
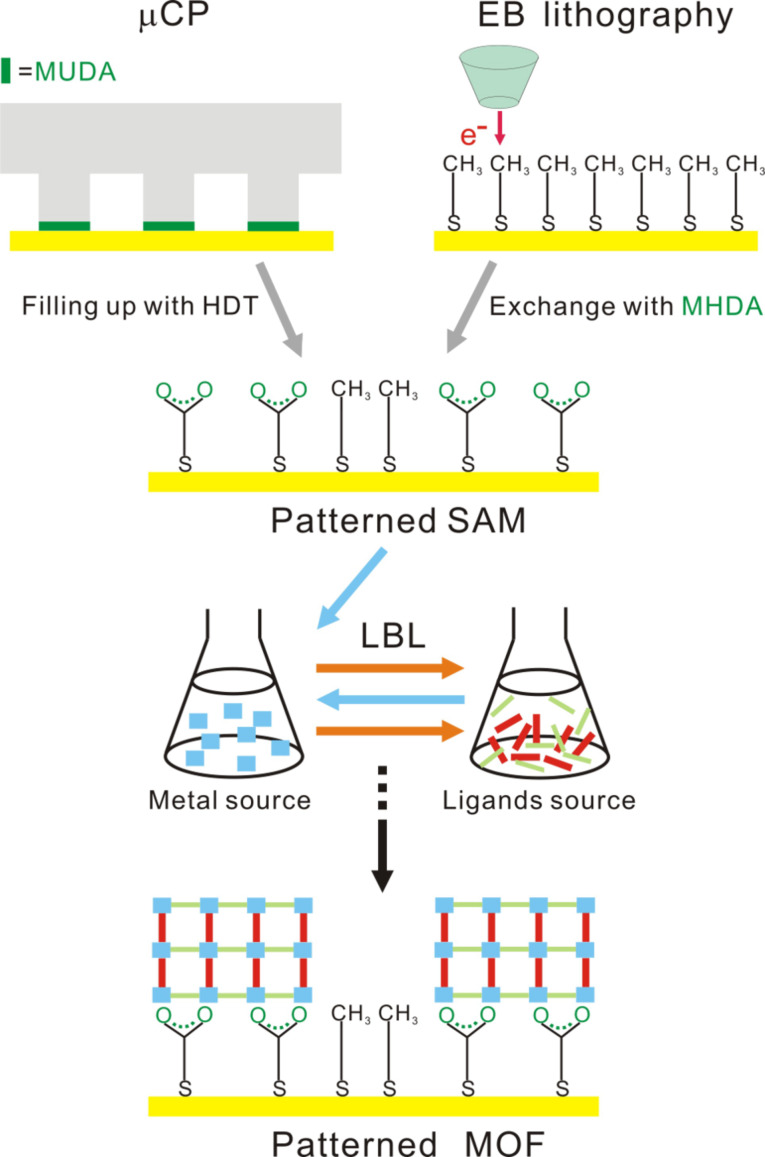
SAMs can be either patterned by µCP (top row, left) or by EB lithography (top row, right) before the layer-by-layer deposition of [Zn_2_(adc)_2_(dabco)] (bottom).

SAMs cannot only control the crystallographic orientation of SURMOFs, as has been demonstrated above, but in combination with micro/nanofabrication techniques also the lateral control of SURMOF growth is possible, opening valuable opportunities, e.g., for MOF sensor development. Here, we wish to present two different approaches to pattern SAMs, which have been used for the localized growth of SURMOFs. As described in Figure 6, the substrates became patterned with two different functional SAMs, one of which could promote the nucleation (such as a –COOH group), while the other inhibits the nucleation (such as a –CH_3_ group).

**Figure 7:**
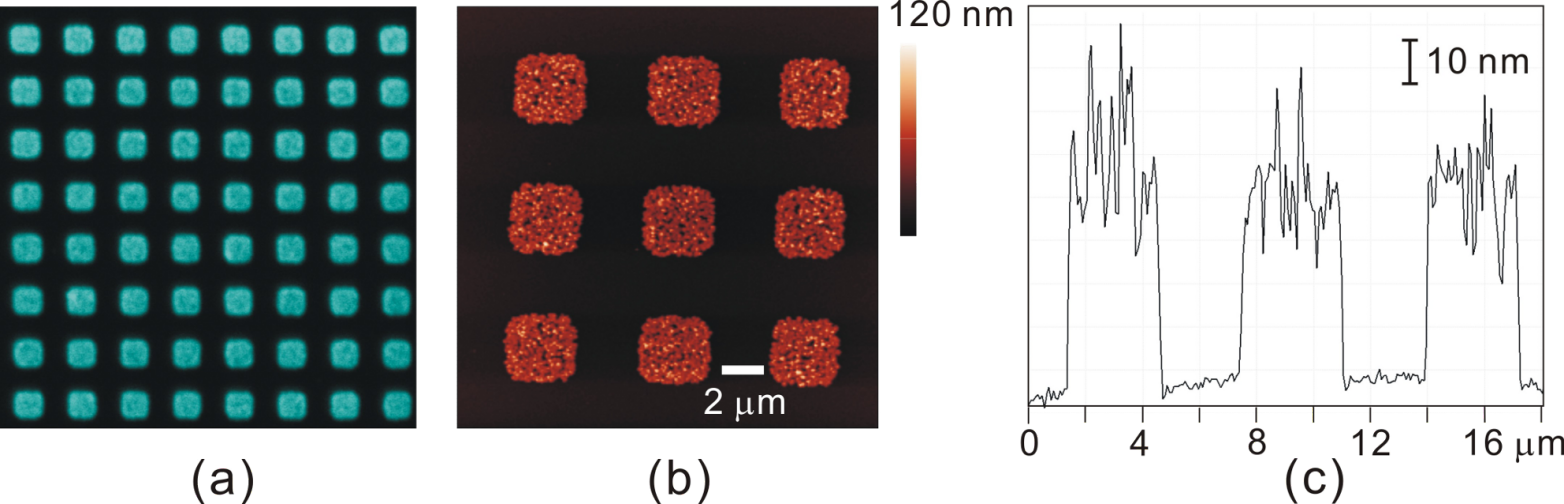
[Zn_2_(adc)_2_(dabco)] patterns grown on MUDA/HDT surfaces (square areas: MUDA, remaining area: HDT) structured by µCP: (a) Epifluorescence image (b) AFM image and (c) AFM cross-sectional profile.

One of the methods to create a patterned SAMs is microcontact printing (μCP) [49]. For this, a microstructured polydimethylsiloxane (PDMS) stamp was inked with 11-mercaptoundecanoic acid (MUDA) to transfer a pattern of 3 µm squares to the Au surface. The area surrounding the MUDA patterned parts was filled with 1-hexadecanethiol (HDT) by simple immersion in its ethanolic solution. Due to the chemical properties of –COOH and –CH_3_, we could expect that the growth of [Zn_2_(adc)_2_(dabco)] would be restricted to the –COOH functionalized areas. As the fluorescence-microscopy image given in Figure 7a demonstrates, the growth of [Zn_2_(adc)_2_(dabco)] on such a patterned surface occurs indeed selectively on the MUDA-functionalized surface (square areas), while the HDT surface shows remarkable inhibition of [Zn_2_(adc)_2_(dabco)] nucleation.

The AFM measurement (Figure 7b,c) shows more details of the SURMOF pattern. In contrast to the nonpatterned MTCA surface, the [Zn_2_(adc)_2_(dabco)] nanocrystals completely cover the MUDA areas, forming a closed, thin film. The average thickness of these films estimated from AFM cross section is about 55 nm after 45 cycles. We believe that the difference in the morphology of [Zn_2_(adc)_2_(dabco)] nanocrystals on both carboxylate (MTCA versus MUDA) terminated surfaces results from the inferior order quality of the MUDA films [40], which causes multiple nucleation sites, from which smaller, but more densely packed MOF crystals grow.

**Figure 8:**
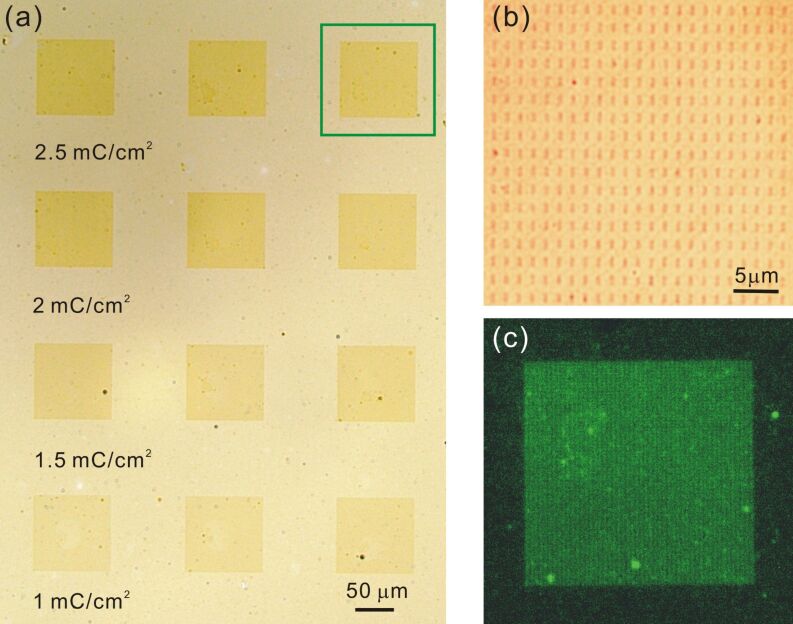
Patterned deposition of [Zn_2_(adc)_2_(dabco)] by using the irradiation-promoted exchange reaction (IPER) to modify the surface chemistry of the SAM. (a) Square arrays of rectangles (1 × 0.3 µm^2^) were written into the HDT SAM by using different area doses (increasing from bottom to top), followed by exchange with MHDA, before the layer-by-layer growth of the MOF was performed. (b) Close-up of one of the arrays (area dose: 2.5 mC/cm^2^) showing the small rectangular deposits of [Zn_2_(adc)_2_(dabco)]. (c) Epifluorescence image (excitation wavelength 475 nm) of the region marked by the green square in (a).

Based on the observation that the exchange reaction of alkanethiol SAMs upon immersion into another ω-substituted alkanethiol solution can be significantly enhanced by electron irradiation, a new patterning technique was developed by Zharnikov et al. [50–54]. Thus, our second strategy to fabricate a patterned SAM was the combination of this irradiation-promoted exchange reaction (IPER) with electron-beam lithography (EB lithography). The primary advantage of EB lithography is that the fabricated features can be in the nanometer regime. Figure 6 (upper right) illustrates the process: Starting from a HDT SAM as the primary matrix, disordering and fragmentation occur within the electron-beam-exposed areas, significantly enhancing the exchange rate during the following immersion into the 16-mercaptohexadecanoic acid (MHDA) solution. These carboxyl-terminated areas then acted as nucleation sites for the growth of the SURMOF, again by the layer-by-layer method. As shown in the optical micrograph in Figure 8a, the irradiated areas indeed became covered with [Zn_2_(adc)_2_(dabco)].

We also observed that with increasing area dose, the growth of [Zn_2_(adc)_2_(dabco)] became more pronounced. Obviously with higher doses, more MHDA molecules become exchanged into the HDT matrix, thus promoting the [Zn_2_(adc)_2_(dabco)] nucleation. The close-up image shows the well-defined rectangles (1 × 0.3 µm^2^) where EB irradiation was performed. Again, the fluorescence microscopy image (Figure 8c) supports the notion that the darker areas in Figure 8a are in fact arrays of rectangles formed by the [Zn_2_(adc)_2_(dabco)] MOF.

## Conclusion

Fluorescent, porous materials, the photoluminescent properties of which are sensitive to certain molecular guests, are promising sensor materials. Nevertheless, their application typically requires some kind of directed immobilization, either regarding their crystallographic orientation, which also determines the orientation and accessibility of their pores, or their lateral distribution, which is a necessity for the fabrication of, e.g., sensor arrays. In this project, we demonstrated the feasibility of self-assembled monolayers for both purposes, since these monolayers are able to interact strongly with the building blocks of the MOFs. In conjunction with the recently established layer-by-layer deposition method, this directed interaction also orients the building blocks during the MOF formation, resulting in well-oriented SURMOF surfaces. When the functional headgroups of the SAM constituents are not distributed evenly over the surface, but are instead patterned, they also direct the location of SURMOF deposition. Two different methods have been employed for the patterning, namely microcontact printing and electron-beam lithography. We demonstrated that both methods provide the possibility to grow SURMOF arrays selectively with micrometer resolution or better. In continuation of this project, we will explore how the orientation of the SURMOFs and their patterning influence the response (selectivity and kinetics) to different analytes.

## Experimental

### Synthesis of anthracene-9,10-dicarboxylic acid (H_2_adc)

The synthesis of 9,10-anthracenedicarboxylic acid mainly followed the procedure described in the literature [55]. *n*-Butyllithium (22.5 mmol, 1.44 g) was added under nitrogen to a suspension of 9,10-dibromoanthracene (7.5 mmol, 2.50 g) in absolute diethyl ether (30 mL). The reaction mixture was stirred for 20 min and a colour change from yellow to dark yellow indicated the formation of the dilithium compound. Dry ice (approximately 10 g) was added to the mixture and stirred until the excess evaporated. After the addition of water (100 mL) and diethyl ether (75 mL), the aqueous phase was separated. Diluted sulfuric acid (H_2_SO_4_, 3%, 40 mL) was added and an immediate precipitation of yellow crystals was observed. The crude product was collected by filtration and washed with cold water, and then dried overnight in an oven. Recrystallization in diluted ethanol (10%) could not completely remove the impurities (mainly 9-anthracenecarboxylic acid). Therefore, the crude product was purified by gradient sublimation at 250 °C at a pressure of 10^−5^ mbar. ^1^H NMR (DMSO-*d*_6_, 300 MHz) δ 7.66–7.72 (m, 4H), 8.04–8.10 (m, 4H), 14.14 (br, 2H). Anal. calcd for C, 72.10; H, 3.75; found C, 72.00; H, 4.00.

### Synthesis of bulk [Zn_2_(adc)_2_(dabco)] as a reference material

[Zn_2_(adc)_2_(dabco)] crystals were prepared according to the literature procedure [36]. Zn(NO_3_)_2_·6H_2_O (0.30 g, 1 mmol) and H_2_adc (0.27 g, 1 mmol) were dissolved in DMF (5 mL). A solution of dabco (0.06 g, 0.5 mmol) in MeOH (5 mL) was added to the mixture under stirring for 12 h at room temperature. The white precipitate formed was filtered off. The resulting clear solution was heated to 120 °C for 2 d. The colourless crystals were collected and washed with DMF, MeOH and dried in an oven.

### Synthesis of [Zn_2_(adc)_2_(dabco)] nanocrystals

In contrast to the bulk synthesis, no heating was applied. For example, 5 mL of a DMF solution of an equimolar mixture of Zn(NO_3_)_2_·6H_2_O and H_2_adc, respectively, (”Zn-adc”, 50 mM or 25 mM each) was mixed with 5 mL of a 50 mM solution of dabco in methanol under stirring for 15 min at room temperature. Within a few minutes, the mixture turned to a suspension. The precipitate was collected by centrifugation and washed with methanol twice. Finally, the precipitate was dried under vacuum. Yield: 0.13 g. By changing the concentration of the precursor solutions, the other nanocrystal samples were synthesized. The yield for Zn–adc/dabco = 25 mM:25 mM was 0.05 g and for Zn–adc/dabco = 25 mM:12.5 mM was 6 mg.

### SAM-functionalized substrates

The Au substrates were manufactured by electron-beam evaporation of 5 nm of Cr and 100 nm of Au onto four-inch Si wafers with (100) orientation. Whenever these films could not be used immediately, they were cleaned prior to use by immersion into a 10 mM 1-hexadecanethiol (HDT, Aldrich) solution in ethanol for 2 h followed by a 2 min treatment in H_2_ plasma [56]. The clean gold substrates were immersed either in a 0.1 mM (4'-(pyridin-4-yl)-[1,1-biphenyl]-4-yl)methanethiol (PPP1, synthesized according to [57]) or 0.1 mM 4'-(mercaptomethyl)-terphenyl-4-carboxylic acid (MTCA, synthesized according to [40]) solution in ethanol for 24 h.

### Microcontact printing

Patterned SAMs were fabricated by microcontact printing (µCP) using PDMS stamps, which were cast from a master fabricated by photolithography. The pattern consisted of an array of 3 µm protruding squares with a distance of 3 µm. The stamps were inked with 11-mercaptoundecanoic acid (MUDA, 3 mM solutions in ethanol) and brought into contact with the Au surface for 20 s. The resulting patterned Au substrates were immersed in HDT solution (10 mM in ethanol) for 5 min, and then washed with ethanol followed by drying in a stream of N_2_.

### Electron-beam lithography

The cleaned Au substrate was immersed into a 10 mM 1-hexadecanethiol (HDT, Aldrich) solution in ethanol for 4 h. After being rinsed with ethanol, and dried with N_2_, the sample was ready for e-beam writing. The sample was e-beam patterned by a JEOL JSM-7001F scanning electron microscope, equipped with a XENOS XeDraw2 lithography system. The beam current was 200 pA and the acceleration voltage 15 kV. Area doses were varied between 0.1 and 2.5 mC/cm^2^.

### Layer-by-layer growth of [Zn_2_(adc)_2_(dabco)] on SAM-functionalized surfaces

Layer-by-layer deposition was performed in a custom-made, temperature-controllable glass cell. The functionalized substrates were alternately immersed into a zinc acetate dihydrate solution in ethanol (1 mM) for 20 min and in an equimolar H_2_adc/dabco mixture (0.1 mM each) for 40 min. Between each step, the substrate was purged with fresh ethanol for 5 min twice.

### Characterization

SEM images were recorded on a JEOL JSM 7001F scanning electron microscope. Powder X-ray diffraction patterns were collected between 2θ = 2 and 90°, on a STOE theta/theta diffractometer by using Cu Kα_I_ (1.5418 Å) radiation and a linear position-sensitive detector. The surface X-ray diffraction (SXRD) measurements were performed in theta/theta mode, with a step width of 0.02°, and a scan rate of 100 s per step for thin-film samples. AFM measurements were performed on a NanoScope Dimension^TM^ 3100 atomic force microscope in tapping mode. FT-IR spectra were recorded with a NICOLET 6700 Fourier transform infrared reflection–absorption spectrometer. For bulk substances a diamond ATR cell was used; for thin films on reflective substrates (gold) a modified smart SAGA unit providing an incidence angle of 80° was utilized. SAMs of perdeuterated hexadecanethiol (C_16_D_33_SH) on gold were used as background samples for the thin-film FT-IR measurement. Photoluminescence spectra were recorded on a PerkinElmer LS 50B fluorescence spectrometer. Epifluorescence images were recorded on an Olympus BX51 fluorescence system. Laser scanning confocal microscopy (LSCM) was carried out on a Zeiss LSM 510 META microscope.
